# Complete Genome Sequence of *Leptospira interrogans* Strains FMAS_KW1, FMAS_KW2 and FMAS_AW1 Isolated from Leptospirosis Patients from Karawanalla and Awissawella, Sri Lanka

**DOI:** 10.7150/jgen.43953

**Published:** 2020-04-22

**Authors:** Indika Senevirathna, Dinesha Jayasundara, Joshua P. Lefler, Kira L. Chaiboonm, Janith Warnasekara, Suneth Agampodi, Michael A. Matthias, Joseph M. Vinetz

**Affiliations:** 1 Leptospirosis Research Laboratory, Department of Community Medicine, Faculty of Medicine and Allied Sciences, Rajarata University of Sri Lanka; 2 Department of Biochemistry, Faculty of Medicine and Allied Sciences, Rajarata University of Sri Lanka; 3 Department of Microbiology, Faculty of Medicine and Allied Sciences, Rajarata University of Sri Lanka; 4 Department of Medicine. Division of Infectious Diseases, University of California, San Diego. California, United States of America.; 5 Department of Community Medicine, Faculty of Medicine and Allied Sciences, Rajarata University of Sri Lanka; 6 Section of Infectious Disease, Department of Internal Medicine, School of Medicine, Yale University, New Haven, CT, USA

**Keywords:** Leptospira, Leptospirosis, Sri Lanka, Full Genome

## Abstract

Leptospirosis is an important cause of acute undifferentiated fever and complex multisystem febrile diseases in the tropics and subtropics. Understanding the evolution of *Leptospira* especially as related to the clinical pathogenesis of leptospirosis is facilitated by systematic comparative genomic analysis of human-infecting isolates. Here, we announce the complete genome sequences of three *Leptospira* strains that were isolated from blood of humans with undifferentiated fever in Sri Lanka.

## Introduction

Leptospirosis is a globally-distributed, potentially-fatal, emerging infectious disease 1. Case fatality rates of up to 20% are due to inadequate diagnostic tools, a limited understanding of mechanisms of disease pathogenesis, and poorly understood leptospiral virulence mechanisms. Symptoms vary from a self-resolving, undifferentiated febrile illness to multiorgan failure and fulminant death. *Leptospira* are genetically- and antigenically-diverse Gram-negative spirochetes phylogenetically-resolved into three sub-clades of six or more species of similar pathogenicity, and 24 well-studied serogroups [>300 distinct serotypes]. Until recently, the varied clinical presentation of leptospirosis was a presumed to be related to leptospiral diversity with certain species and serovars being inherently more virulent, but evidence to support this assumption is not supported by comparative whole genome analysis (CWGA)^2^. *Leptospira* CWGA is based on long-term culture-adapted strains [non-pathogens, attenuated clinical isolates, and avirulent reference strains]; most are based on draft genomes (https://www.ncbi.nlm.nih.gov/genome/genomes/). Consequently, genome data from low-passage strains are critical. Here, we describe the annotation of recently-completed genomes of *L. interrogans* strains [FMAS_KW1, FMAS_KW2 and FMAS_AW1] recently isolated from three leptospirosis patients.

All strains were isolated in the context of a prospective clinical study of undifferentiated febrile illness in humans that began in 2015 from epidemiologically-contrasting sites around Sri Lanka. Full details of the study and methods have been published elsewhere 3. Samples for cultures were obtained from febrile patients who were clinically classified as 'probable' leptospirosis cases who presented at hospital, two from Karawanalla (FMAS_KW1 and FMAS_KW2), and one from Awissawella (FMAS_AW1). At the bedside, four drops of fresh whole blood were inoculated into 9 mL of semisolid EMJH 4 medium supplemented with 5-flourouracil and neomycin, incubated at 30^0^C, and checked biweekly under darkfield microscopy for growth. All three isolates grew slowly, requiring 10, 15 and 17 weeks post-inoculation, respectively, before cells were visible. Serotyping of newly isolated *Leptospira* strains was done in the Pasteur Institute, France, using a standard panel of rabbit antisera against reference serovars representing 24 main serogroups 5. Semisolid cultures were sub-cultured into liquid EMJH medium, and sub-cultured no more than twice more in liquid EMJH prior to genomic DNA extraction, which was done from log phase growth^6^; DNA purification was done using a commercially available genomic DNA purification kit (Gene Jet, ThermoFisher). SMRTbell libraries were generated and sequenced on a PacBio RS II system (Pacific Biosciences, Menlo Park, CA, USA). A minimum of 30X read coverage was obtained for all three isolates. Raw read data were preprocessed using an in-house developed quality control pipeline. Genomes were assembled *de novo* using Canu 1.8 7 then circularized using Circlator 8 (http://sanger-pathogens.github.io/circlator), and polished using Quiver 9. The workflow resulted in two, three and four overlapping contigs each for FMAS_KW2, FMAS_KW1 and FMAS_AW1, respectively. The fully closed genomes were then annotated by the NCBI Prokaryotic Genome Annotation Pipeline. Software was run with default settings.

Genome sizes ranged from 4.65Mbp (FMAS_KW2) to 5.07 Mbp (FMAS_AW1), Table 1. All contained the typical two chromosomes, and all encoded 37tRNA genes covering all 20 amino acids (Table 1). Two strains, FMAS_AW1 and FMAS_KW1, contained additional large replicons presumptively classified as plasmids by curators of the NCBI Prokaryotic Genome Annotation Pipeline resource (Table 1). Notably, FMAS_AW1 is amongst the largest *Leptospira* genomes reported to date, with its presumed plasmid, pLiSL1 (approx. size, 130 Kbp), being the largest known extra-chromosomal *Leptospira* replicon.

**Figure 1:**
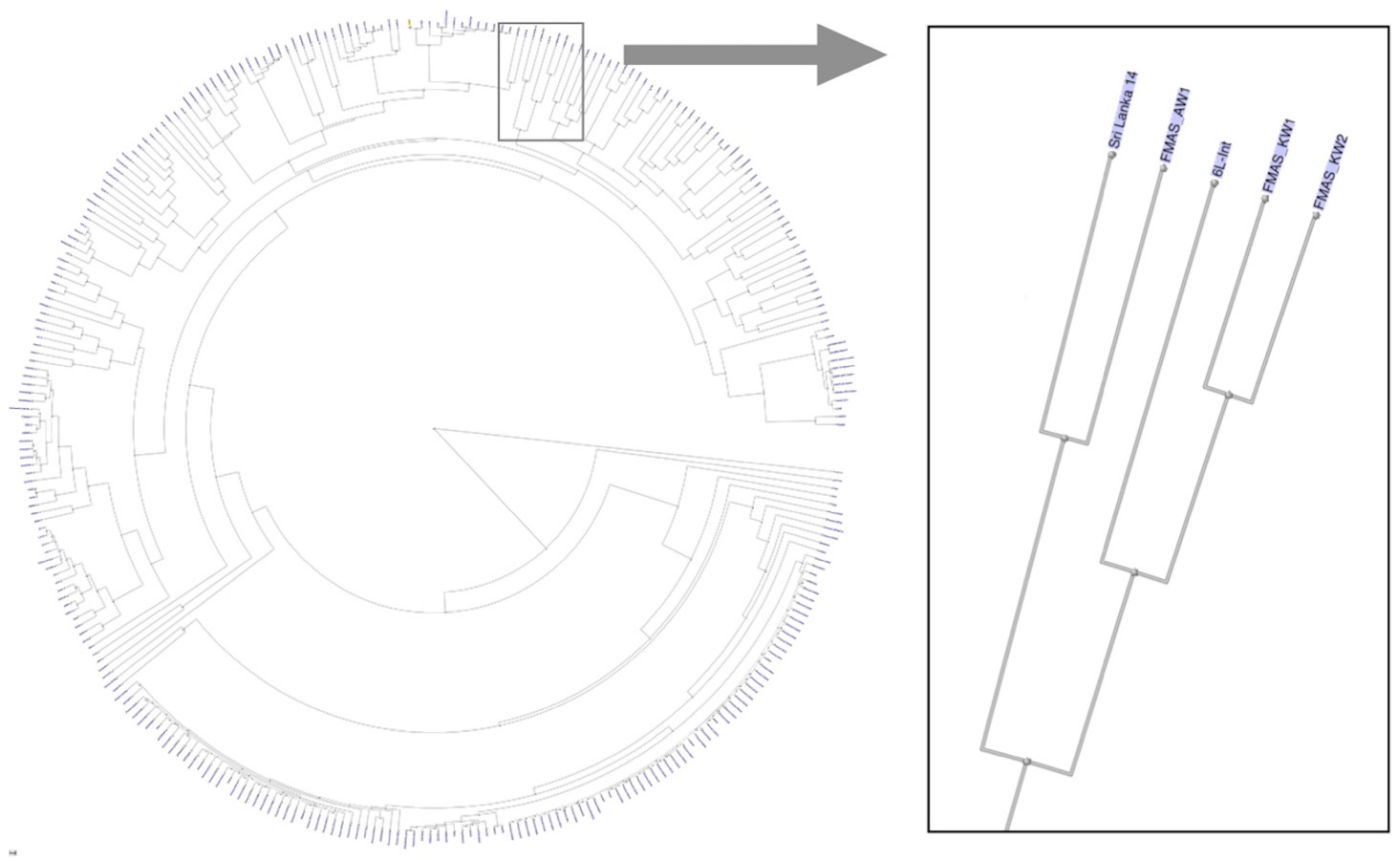
Dendrogram based on genomic blast against the NCBI whole genome database consisted 311 *L. interrogans* genomes, showing the newly isolated strains of Leptospira spp. from Sri Lanka.

The dendrogram based on genomic blast against the NCBI whole genome database (https://www.ncbi.nlm.nih.gov/genome/179?) including 311 *L. interrogans* genomes shows that FMAS_KW1, FMAS_KW2 and FMAS_AW1 form a discrete cluster with two previously sequenced strains: *L. interrogans* serogroup Autumnalis serovar Weerasinghe strain 6L-int, and *L. interrogans* serogroup Pyrogenes serovar Pyrogenes strain Sri Lanka 14 (Figure 1), both of which have been isolated previously in Sri Lanka^10^. Serotyping data from FMAS_KW2 and FMAS_AW1 showed that are within serogroup Autumnalis. FMAS_KW1 was slow growing and was unable to be serotyped in the reference laboratory.

Five leptospiral species--*L. interrogans, L. kirschneri, L. borgpetersenii, L. weilli* and* L. santarosai*--have been reported as causing human leptospirosis in Sri Lanka 10. The strains from which this information derived is from Sri Lanka dating to the 1960s and 1970s, except the two strains reported in 2018 11, showing current scarcity of knowledge on circulating strains of *Leptospira* in Sri Lanka. Of these, several annotated, genomes originating from Sri Lanka of strains of imprecise provenance and very large passage number are publicly available. Of leptospiral species/strains isolated in Sri Lanka to date, only the genome of *L. borgpetersenii* serogroup Sejroe serovar Ceylonica strain Piyasena isolated in 1964 (from a male patient in Colombo) has been completed and closed. The present work adds three more complete genomes to this database, widening the knowledge on *Leptospira* genome. In-depth analysis of new genomes published in the present paper will enhance the knowledge on pathogenesis and evolution of *Leptospira*.

## Figures and Tables

**Table 1:** Genome architecture of two *Leptospira* isolates from Sri Lanka

Strain	Type	Name	Size (Mb)	GC%	Protein	rRNA	tRNA	Other RNA	Gene	Pseudogene	RefSeq	INSDC	
FMAS_KW1	Chr	I	4.31	35	3,271	5	37	2	3,529	214	NZ_CP039258.1	CP039258.1	https://www.ncbi.nlm.nih.gov/nuccore/CP039258.1
	Chr	II	0.36	35	283	-	-	-	294	11	NZ_CP039259.1	CP039259.1	https://www.ncbi.nlm.nih.gov/nuccore/CP039259.1
	Plsm	pLiLS1	0.08	34.3	63	-	-	-	84	21	NZ_CP039260.1	CP039260.1	https://www.ncbi.nlm.nih.gov/nuccore/CP039260.1
FMAS_KW2	Chr	I	4.29	35	3,318	5	37	2	3,542	180	NZ_CP039256.1	CP039256.1	https://www.ncbi.nlm.nih.gov/nuccore/CP039256.1
	Chr	II	0.36	35	284	-	-	-	294	10	NZ_CP039257.1	CP039257.1	https://www.ncbi.nlm.nih.gov/nuccore/CP039257.1
FMAS_AW1	Chr	I	4.5	35.2	3,491	5	37	2	3,735	200	NZ_CP039283.1	CP039283.1	https://www.ncbi.nlm.nih.gov/nuccore/CP039283.1
	Chr	II	0.36	35.1	292	-	-	-	299	7	NZ_CP039284.1	CP039284.1	https://www.ncbi.nlm.nih.gov/nuccore/CP039284.1
	Plsm	pLiSL1	0.13	35.2	126	-	-	-	137	11	NZ_CP039285.1	CP039285.1	https://www.ncbi.nlm.nih.gov/nuccore/CP039285.1
	Plsm	pLiSL2	0.08	34.3	65	-	-	-	84	19	NZ_CP039286.1	CP039286.1	https://www.ncbi.nlm.nih.gov/nuccore/CP039286.1

## References

[1] Costa F, Hagan JE, Calcagno J, Kane M, Torgerson P, Martinez-Silveira MS (2015). Global Morbidity and Mortality of Leptospirosis: A Systematic Review. PLoS Negl Trop Dis.

[2] Guglielmini J, Bourhy P, Schiettekatte O, Zinini F, Brisse S, Picardeau M (2019). Genus-wide Leptospira core genome multilocus sequence typing for strain taxonomy and global surveillance. PLoS Negl Trop Dis.

[3] Agampodi S, Warnasekara J, Jayasundara D, Senawirathna I, Gamage C, Kularatne S (2019). Study protocol: characterising the clinical, epidemiological and aetiological aspects of leptospirosis in Sri Lanka: a hospital based clinico-epidemiological study. BMJ open.

[4] Bey RF, Johnson RC (1978). Protein-free and low-protein media for the cultivation of Leptospira. Infect Immun.

[5] Bourhy P, Collet L, Clement S, Huerre M, Ave P, Giry C (2010). Isolation and characterization of new Leptospira genotypes from patients in Mayotte (Indian Ocean). PLoS Negl Trop Dis.

[6] Fouts DE, Matthias MA, Adhikarla H, Adler B, Amorim-Santos L, Berg DE (2016). What Makes a Bacterial Species Pathogenic?:Comparative Genomic Analysis of the Genus Leptospira. PLoS Negl Trop Dis.

[7] Koren S, Walenz BP, Berlin K, Miller JR, Bergman NH, Phillippy AM (2017). Canu: scalable and accurate long-read assembly via adaptive k-mer weighting and repeat separation. Genome Res.

[8] Hunt M, Silva ND, Otto TD, Parkhill J, Keane JA, Harris SR (2015). Circlator: automated circularization of genome assemblies using long sequencing reads. Genome Biol.

[9] Chin C-S, Alexander DH, Marks P, Klammer AA, Drake J, Heiner C (2013). Nonhybrid, finished microbial genome assemblies from long-read SMRT sequencing data. Nature methods.

[10] Naotunna C, Agampodi SB, Agampodi TC (2016). Etiological agents causing leptospirosis in Sri Lanka: A review. Asian Pac J Trop Med.

[11] Nisansala G, Muthusinghe D, Gunasekara T, Weerasekera M, Fernando S, Ranasinghe K (2018). Isolation and characterization of Leptospira interrogans from two patients with leptospirosis in Western Province, Sri Lanka. Journal of medical microbiology.

